# Dynamic nature of viral and bacterial communities in human faeces

**DOI:** 10.1016/j.isci.2023.108778

**Published:** 2023-12-29

**Authors:** Andrey N. Shkoporov, Orla O'Regan, Linda Smith, Ekaterina V. Khokhlova, Lorraine A. Draper, R. Paul Ross, Colin Hill

**Affiliations:** 1 APC Microbiome Ireland, University College Cork, Cork, Ireland; 2 School of Microbiology, University College Cork, Cork, Ireland; 3 Department of Medicine, University College Cork, Cork, Ireland

**Keywords:** virology, microbiology, microbiome

## Abstract

Bacteriophages are a major component of the gut microbiome and are believed to play a role in establishment and stabilization of microbial communities by influencing taxonomic and functional diversity. We show that the activity of lytic and temperate phages can also significantly affect bacterial community structure in a model of extended colonic retention. Intact fresh human feces were incubated anaerobically at 37°C without homogenization and subjected to metagenomic sequencing. We observed subject-specific blooms and collapses of selected bacteriophage and bacterial populations within some individuals. Most notable were striking collapses of *Prevotella* populations accompanied by increases in specific bacteriophages. In a number of cases, we even observed a shift from one bacterial “enterotype” to another within 48 h. These results confirm that intact feces represents a highly dynamic ecological system and suggests that colonic retention time could have a profound effect on microbiome composition, including a significant impact by bacteriophages.

## Highlights

• Significant changes occur in human fecal bacteriome and virome during *ex vivo* incubation
• Specific phage and bacterial dynamics are dependent on microbiome context
• Collapse of Prevotella population is mirrored by an increase of Prevotella phages
• Incubation of fecal microbiome can result in changes comparable to a switch of enterotype

## Graphical abstract

**Figure:**
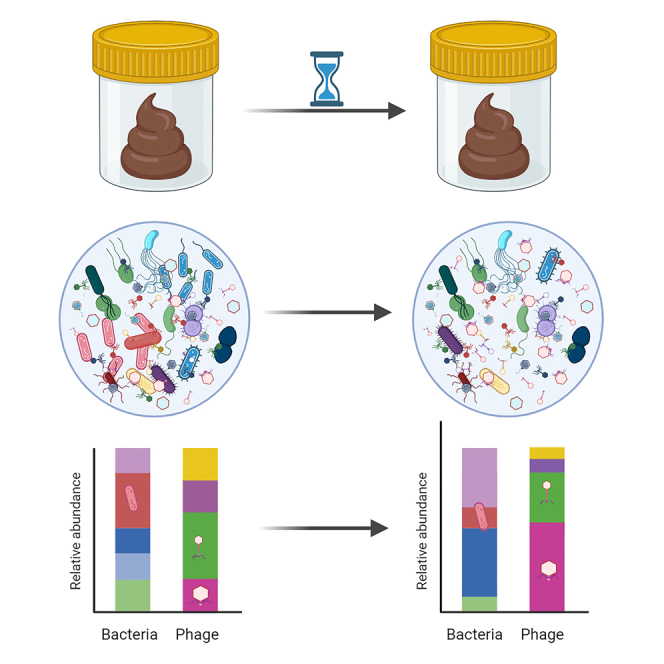

## Introduction

Human gut microbiome composition is highly individualized,^1^^,^^2^ which makes it difficult to define a “healthy” gut microbiome and complicates the analysis of compositional changes in microbiota in cohorts associated with different diseases.^3^ It has also been shown that microbiome individuality can underpin individual-specific responses to diet^4^ and medications.^5^ Inter-individual microbiome variation represents a continuum of states, defined by gradients of concentration of the predominant taxa.^6^ The concept of “enterotypes”, or a fixed number of poles (usually three, defined by the dominance of *Bacteroides* and *Prevotella* genera, or the *Ruminococcaceae* family^7^) to which different intermediate states seem to gravitate, has been used to interpret gut microbiome data. The analysis of the human gut microbiome is further complicated by significant radial and longitudinal variation throughout the anatomy of the gastrointestinal tract^8^ and reliance on fecal samples as faithful proxies for the distal gut microbiome.

While factors responsible for driving the composition and stability of individual gut microbiomes are poorly understood, large population analyses of gut microbiota variation in healthy subjects have suggested several abiotic co-variates, most importantly stool consistency and moisture content linked to colonic transit time, that strongly correlate with microbiome composition, enterotype and total microbial loads.^9^^,^^10^^,^^11^^,^^12^ In particular, it has been demonstrated that looser stools with higher moisture content tend to display decreased microbial diversity. For *Bacteroides* and *Ruminococcaceae*-dominated enterotypes the *Bacteroides*:*Ruminococcaceae* ratio increases with increasing stool water content. It has been suggested that the relatively faster growth rates of *Bacteroides* can counter their washout at higher gut transit rates. At the same time, firmer stools contain increased quantities of *Akkermansia*, and the methane-producing archaeon *Methanobrevibacter*, as well as display a shift from carbohydrate fermentation to protein catabolism in microbial energy generation.^13^ Interestingly, the enterotype dominated by slow-growing *Prevotella* is associated with diets rich in plant fiber,^14^ shorter transit time, and lower total microbial loads.^12^ This enterotype does not show any tendency for the enrichment of fast growers, suggesting that a different strategy is used by *Prevotella* (such as stronger adherence to mucus and epithelia) to avoid washout.^11^ This concept holds true when tested *in vitro* (in a three-stage continuous culture system), where increase in transit time resulted in decrease of biomass and diversity in the distal compartments.^15^

In addition to abiotic properties of the microbial habitat (such as availability and type of nutrients, peristaltic rate, etc.), the role of biotic factors must be taken into account. Bacteriophages (phages) are one of the prominent biotic forces operating in the gut microbiome with potential for exerting control over diversity and composition of the bacteriome. Advances in sequencing technology have paved the way for metagenomic studies that have helped to uncover the extraordinary diversity of largely uncultured and unclassified phage populations in the gut—the virome.^16^ Phages are as abundant as bacterial cells, reaching 10^11^ virus-like particles (VLP) g^−1^,^17^ and one would predict that such a mass of bacteriophages would exert a strong top-down control on the density and diversity of the bacteriome.^18^^,^^19^ However, the majority of these particles belong to viruses that either have been predicted to have a temperate lifestyle,^20^ or have been shown to undergo lytic replication in restricted sub-populations of their hosts, both factors that would limit phage-induced mortality in bacterial hosts.^21^^,^^22^ Nevertheless, it is conceivable that even when microbiota shifts are driven largely by abiotic factors such as changes in colonic retention time (diarrhea or constipation), phages can act as immediate effectors responsible for bacterial mortality, opening the way for replacement of bacterial populations, and changes of overall diversity. The exact mechanisms responsible can range from prophage induction caused by bacterial SOS response, increased susceptibility to virulent phages due to reduced fitness or changes in cell surface structures, or potentially increased accessibility to bacterial prey due to changes in stool consistency.

It is known that feces, despite a high bacterial biomass content and likely near depletion of nutrients, remains a dynamic system with many bacteria, including strict anaerobes, remaining viable,^23^^,^^24^ metabolically active,^25^ and in a state of active DNA replication.^26^ This opens opportunities for dynamic observations of fecal microbiota, including phage-host interactions, using simple *ex vivo* models.^27^ In this study, we simulated extended colonic retention time by anaerobically incubating freshly collected human feces and registering changes in microbial and phage community composition over time. We started from a premise that this extended anaerobic incubation will likely change the composition of fecal microbial communities, in a manner similar to how increased retention time changes the composition of fecal microbiota in the human colon. We also hypothesized that phages (temperate and virulent) might have a direct role in this process, facilitating the demise of certain bacterial populations. Using this simple model we were able to draw parallels with previously observed correlations of microbiota composition and colonic residence time/stool consistency in humans.^11^ Most importantly, we observed correlations between collapse or overgrowth of certain bacterial populations and either collapse or bloom of phage populations.

## Results

### Significant changes in human fecal bacteriome and virome during *ex vivo* incubation

**Figure 1:**
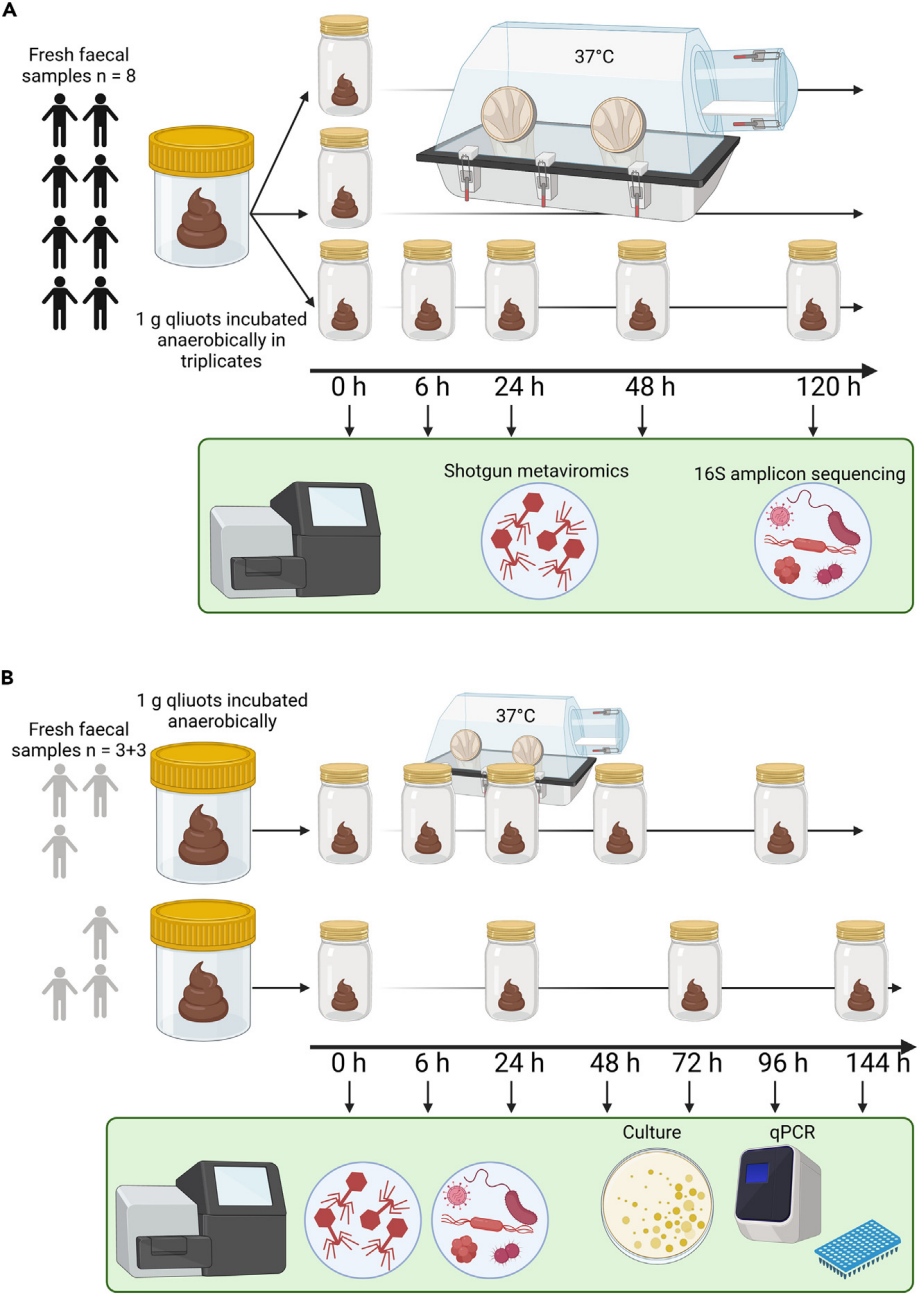
Overview of the experimental design Human fecal samples were collected and subjected to anaerobic incubation at 37°C. (A) Fecal samples from eight subjects were divided into three 1 g aliquots and incubated for 0-6-24-48-120 h, followed by shotgun metaviromics and 16S rRNA amplicon sequencing. (B) Samples from a second group of six subjects were processed similarly but without triplicates and subjected to plating and qPCR assays in addition to sequencing.

Fresh fecal samples were collected from eight healthy adult volunteers (subject codes 916, 920, 922, 923, 926, 928, 941, 943). One gram fecal aliquots were held under anaerobic incubation at 37°C to simulate extended colonic retention time. Three aliquots were removed from each sample at each time point: 0 h, 6 h, 24 h, 48 h, and 120 h of incubation (Figure 1A). In order to assess changes in viral and bacterial diversity and taxonomic composition, shotgun sequencing of DNA extracted from the fecal VLP fraction, as well as sequencing of fecal 16S rRNA gene amplicon libraries, were performed.^28^ Fecal samples collected from six additional subjects were subsequently processed in a similar manner, though without triplicate sampling (Figure 1B).

**Figure 2:**
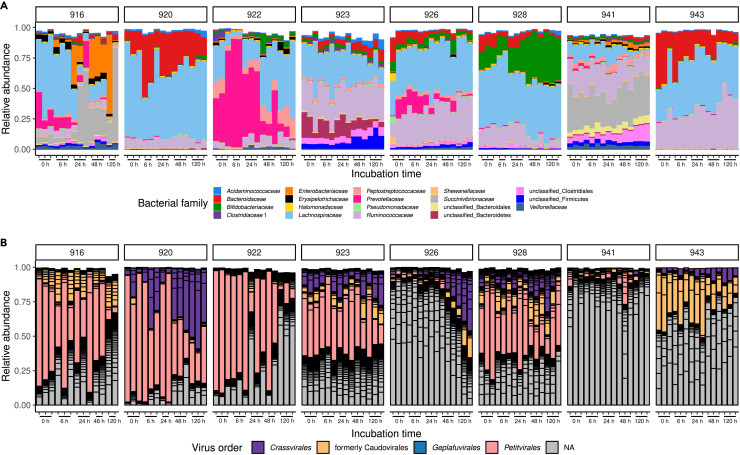
Changes in bacteriome and virome composition during anaerobic incubation of eight human fecal samples (A) Relative abundance of bacterial families reaching at least 5% of read counts in any of the samples. (B) Relative abundance of viral contigs (black outlines) reaching at least 0.1% of read counts in any of the samples. Bars are colored by viral order. All samples were taken as biological triplicates (duplicates in some cases due to failed DNA extraction/sequencing).

The assembly of fecal viromes revealed a total of 12,828 viral genomic contigs across all 14 fecal samples, both partial (12,442) and nearly complete (386) that ranged in size from 1 kb to 196 kb (Figure S1A). Apart from a relatively small fraction of eukaryotic dsDNA and ssDNA viruses present in some of the samples, the DNA virome was dominated by phages belonging to the order *Crassvirales*, other orders and families of the class *Caudoviricetes*, as well as the order *Petitvirales* of small tailless phages (family *Microviridae*, Figure S1). Some of these phage contigs (including 180 out of 386 nearly complete genomes) could be linked to suspected bacterial hosts by means of sequence matches with CRISPR spacer database or sequence similarity to known phages and prophages present in bacterial genomes from RefSeq and HMP reference genome databases. Overall, the taxonomic composition of individual viromes agrees with previous observations.^17^^,^^29^^,^^30^ The bacteriome was represented by 15 dominant families of phyla Bacillota, Bacteroidota, Actinobacteriota, and Pseudomonadota, once again in line with typical fecal bacterial composition (Figure 2A).^7^^,^^12^

Based on the results from the incubation of the initial eight fecal samples, we observed a tendency for an increase in viral contig α-diversity over time, for both Shannon and Simpson indices (Pearson r = 0.34 and 0.28 [weak correlation], p = 0.00024 and 0.0025, respectively, Figure S2). A slight tendency (statistically insignificant at this sample size) for a reduction of bacterial Shannon diversity was also observed. The bacterial community structure assessed at the individual operational taxonomic unit (OTU) level demonstrated significant evolution over time in all samples. Measures of β-diversity between samples, such as Bray-Curtis dissimilarity, showed a steady departure over time from the original viral and bacterial community composition (Pearson r = 0.65 for bacterial community [moderate correlation], p = 5.1e-06; r = 0.76 for viral community, p = 1.4e-08; Figure S3). With regard to bacterial composition, this process was more evident in some samples than in others (Figures 2A and S4). It was striking that samples showing the most drastic changes in bacteriome structure over time (individuals 916, 922) were characterized by an initial dominance followed by a precipitous reduction of *Prevotellaceae* populations during incubation (Figure 2A). This results in dramatic changes, with bacterial structures in some subjects coming to more closely resemble those of other subjects by the end of incubation, rather than their own original bacteriome composition (Figure S4A). While changes in virome composition appear to be equally significant, greater inter-individual variability of phage populations^17^ outweighs virome shifts induced by incubation (Figure S4B). Nevertheless, in parallel with the extinction of the dominant *Prevotella* in samples from individuals 916 and 922, we observed a striking reduction of *Petitvirales* phages^31^ and an increase in unclassified viruses, which results in completely altered bacteriomes and viromes by the end of the incubation (Figure 2B). In the sample from individual 928, the reduction of *Prevotella* populations was paralleled by a relative increase in *Crassvirales* phage^32^ and a reduction of unclassified viral species (Figure 2B). These observations are consistent with phage proliferation in the fecal samples *ex vivo* and a resulting depletion of certain bacterial taxa.

### Specific phage and bacterial dynamics are dependent on microbiome context

**Figure 3:**
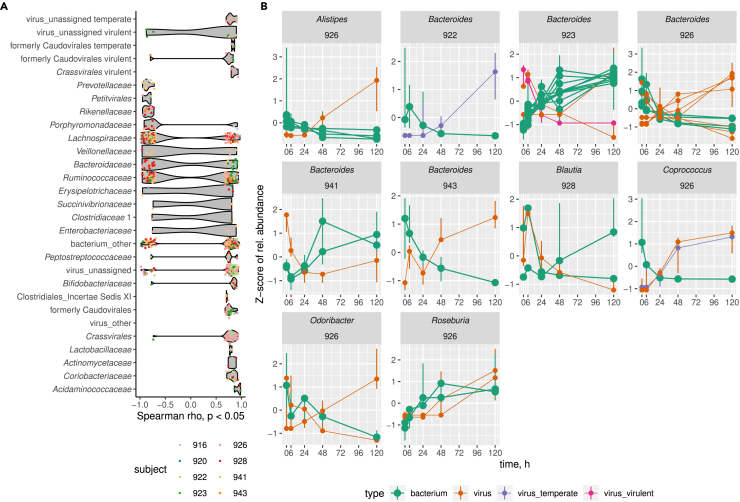
Bacterial OTUs and phage genomic contigs follow different patterns of correlation of their relative abundance with time of incubation (A) Bacterial OTUs (grouped by family) and viral contigs (grouped by viral order) showing positive or negative Spearman rank correlation (p < 0.05) with time within individual fecal samples. (B) Normalized (*Z* score) relative abundance of predicted phage-host pairs (viral contigs linked to bacterial host OTUs via CRISPR spacer matches and inference from closely related phage genomes in IMG/VR3 database. See STAR Methods) showing positive or negative correlation with time of incubation (filled circles are median values, vertical bars show range between minimum and maximum value across three biological replicates).

In order to provide a more detailed view of the dynamics of both the phage and bacterial populations and to identify drivers of microbiome change during incubation, analysis of Spearman rank correlation of fractional abundance of bacterial OTUs and viral genomic contigs against time of incubation was performed on a per subject basis. Each individual fecal sample was characterized by a specific pattern of bacterial OTUs and viral contigs which showed strong positive or negative correlation with the time of incubation (p < 0.05 with Benjamini-Hochberg correction; Figure 3A; Table S3). When correlated viral and bacterial species were grouped to bacterial family and viral order levels, a pattern emerges across individual samples, where the majority of families/orders demonstrate mixed behavior within and across individual fecal samples, with some being either consistently negatively correlated with time (bacterial families *Prevotellaceae*, *Rikenellaceae*, phage order *Petitvirales*) or positively correlated (most of the *Crassvirales* and other class *Caudoviricetes* phages, bacterial families *Acidominococcaceae*, *Actinomycetaceae*, *Coriobacteriaceae*, *Lactobacillaceae*, most of the *Bifidobacteriaceae* and *Peptostreptococcaceae*, Figure 3A). We then focused our analysis on a subset of these time-correlated taxa comprised of possible phage-host pairs (inferred at bacterial genus level) present in the same fecal sample. We observed cases when the normalized relative abundance of bacterial OTUs declined in parallel with increase in relative abundance of the corresponding phage (e.g., *Bacteroides* in individuals 922, 926, and 943; *Coprococcus* and *Odoribacter* in subject 926), and vice versa (e.g., *Bacteroides* in individuals 923 and 941; *Blautia* in subject 928; Figure 3B). Such variable behavior of bacterial strains belonging to the same genera in different contexts can be explained, among other factors, by differences in subject-specific virome composition (both temperate and virulent phages) and differential sensitivity to virulent phage infection or prophage induction in subject-specific bacterial strains.

Taken together, our observations of dramatic changes in both bacterial and viral composition over time in at least some fecal samples incubated anaerobically (Figures 2 and S4), an increase in viral α-diversity (Figure S2A), correlation of specific bacteriome and virome members with time of incubation, and opposite trends in relative abundance of bacteria and their predicted phage, are all consistent with a direct role for phages in bacterial mortality and the concomitant compositional changes of fecal bacteriome. More specifically, observed changes in bacteriome composition can be underpinned by collapses of some bacterial populations caused by virulent phage predation, induction of prophages caused by starvation and other stresses, and overgrowth of other populations, unaffected by phage/prophage attacks. However, other causative factors, such as differential response of microbiota to the inevitable aerobic exposure of fecal samples between sample collection and the beginning of anaerobic incubation, as well as incubation itself, cannot be ruled out.

### Collapse of *Prevotella* population is mirrored by an increase of *Prevotella* phages

Due to the compositional nature of metagenomic data, it is not obvious how the observed changes in relative abundance would translate into changes in absolute abundance of the corresponding bacterial and viral taxa. To clarify that, we proceeded to collect six additional samples from healthy subjects (including one donor from the original eight) to validate our original observations using plate counts, qPCR, and metagenomics. Collected samples were incubated anaerobically in a similar manner, but without triplicates, for either 0-6-24-48-96 h (subjects 925, 927, and 931) or 0-24-72-144 h (subjects 916-1, 921, and 924; Figure 1B).

**Figure 4:**
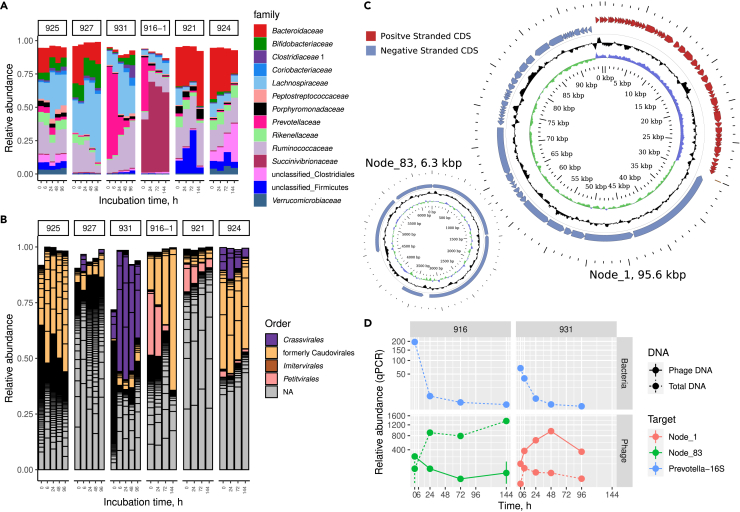
Phages linked to *Prevotella* expand as populations of their hosts collapse in six additionally collected fecal samples (A) Relative abundance of bacterial families reaching at least 5% of read counts in any of the samples. (B) Relative abundance of viral contigs (black outlines) reaching at least 0.1% of read counts in any of the samples. Bars are colored by viral order. (C) Circular maps of phage genomic contigs Node_1 (a *Crassvirales* order phage), Node_83 (a *Petitvirales* order phage). (D) qPCR relative quantification of complete circular phage genomic contigs Node_1, Node_83 and genus *Prevotella* 16S rRNA gene in two fecal samples showing rapid decrease of *Prevotella* populations (filled circles are median values, vertical bars show range between minimum and maximum value across three technical replicates).

This new set of fecal samples displayed dynamics similar to our earlier observations, with dramatic collapses of *Prevotella* populations and changes of virome composition in *Prevotella*-rich samples (Figures 4A and 4B). To further investigate this phenomenon we focused our attention on a group of complete circular viral genomic contigs with proven links to the *Prevotella* genus. These included a 95.6 kb virulent crAss-like phage genome present in sample 931 (exact 34 nt match to a CRISPR spacer GGGTCAGTAGCCATAAGAGTAAATGCAACATCATCAG in *Prevotella copri* strain Indica; Figure 4C), and a 6.3 kb virulent *Microviridae* (order *Petitvirales*) genome present in sample 916-1 (94% and 97% identity matches with CRISPR spacers GACGTATCAGCAGGAGCAGTTTGTTCAGGGGTT and TAATGGAACTATTCTTTATCCTCAATGGGATGAT in *Prevotella copri* strain Indica). Relative quantification of these contigs using qPCR in total community DNA as well as in the VLP-associated fraction of fecal DNA revealed discordant dynamics (Figure 4D). While *Prevotella* levels plummeted in both samples 916-1 and 931, a 95.6 kb crAss-like phage genome, demonstrated a rapid increase that was especially pronounced in the VLP DNA fraction. The 6.3 kb *Microviridae* phage genome sharply increased in the total DNA fraction but unexpectedly showed a slight decrease in the VLP fraction. These results further support the notion that drastic microbiota changes seen in *Prevotella*-rich samples 916-1 and 931 can be attributed to phage activity as one of the contributing factors.

### Incubation of fecal microbiomes can result in changes comparable to a switch of enterotypes

**Figure 5:**
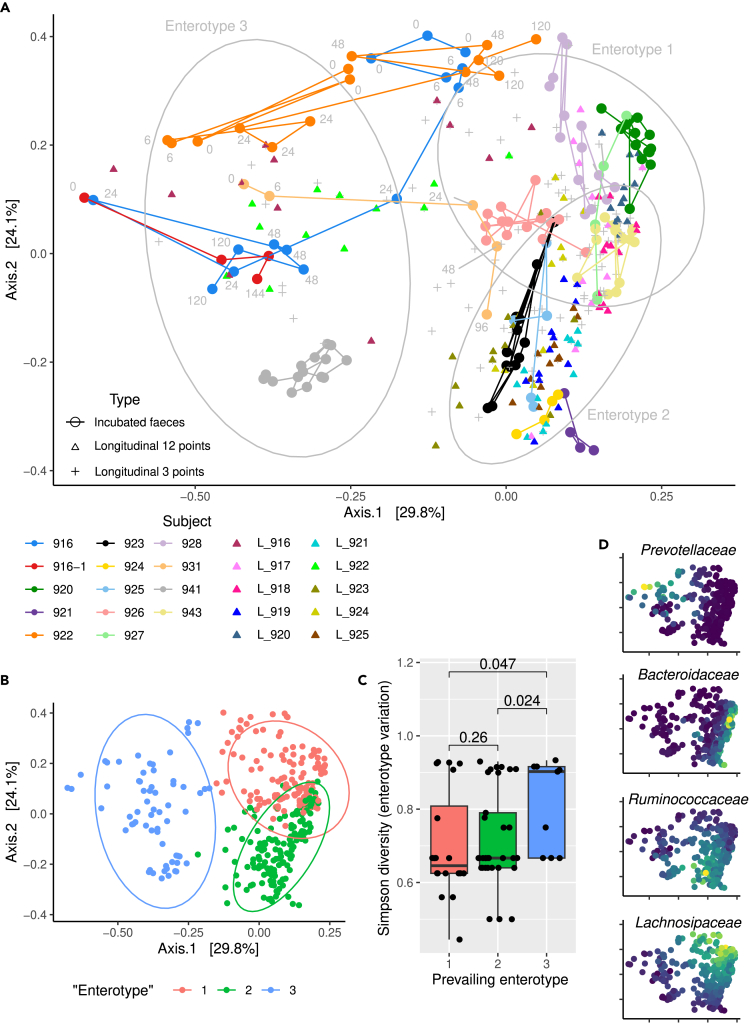
Incubated human fecal samples and samples collected longitudinally over a period of one year can shift “enterotypes” (A) PCoA ordination of Bray-Curtis dissimilarities between incubated samples from the present study (colored circles connected by lines, 13 donors with subject 916 sampled twice, incubation time points are shown for some of the samples), longitudinally collected human fecal samples^17^ (12 monthly samples from ten individuals, colored triangles), and healthy controls (n = 38) from a longitudinal IBD study^33^ (3 samples collected during a year, gray crosses), all samples were processed in the same lab and sequenced using the same protocol (see STAR methods), some donor subjects overlap between these three studies; Confidence ellipsoids represent 95% CI. (B) Bray-Curtis dissimilarities clustered using k-means method into three “enterotypes”. (C) Variation of assigned enterotype within subjects (Simpson’s diversity index) grouped by subjects’ prevailing enterotype (Kruskal-Wallis ranks test p = 0.043, p values of pairwise post hoc Mann-Whitney tests are given in the plot); boxplots are standard Tukey type with interquartile range (box), median (bar) and Q1 – 1.5 × IQR/Q3 + 1.5 × IQR (whiskers). (D) Same PCoA as in A and B overlaid with relative abundance (dark-blue is lower, yellow is higher) of bacterial families *Prevotellaceae*, *Bacteroidaceae*, *Ruminococcaceae*, and *Lachnospiraceae*.

The scale of changes in bacterial communities during incubation prompted us to fit these changes into the context of the “enterotype” concept. To do that, we combined the 16S rRNA sequencing data generated here with data from a year-long longitudinal observation (monthly sampling) of fecal bacteriomes in ten individuals,^17^ as well as longitudinally collected samples (three during a year for each subject, n = 38) from a healthy control cohort of a comparative IBD microbiome/virome study^33^ (Figure 5A). Since all samples were processed in the same lab using the same protocol, and some donor subjects were enrolled in all three studies, we deemed these three datasets directly comparable. K-means clustering of Bray-Curtis dissimilarities between all samples readily separated them into three partially overlapping clusters, or “enterotypes” 1–3 (Figure 5B). A number of incubated fecal samples (916, 922, and 931), as well as longitudinally sampled subjects 916 and 922, migrated from one enterotype to another during the course of the studies (Figure 5A). We then used Simpson diversity index to measure variance of the enterotype label over time within each subject (or each incubated fecal sample). Confirming our observations of instability of *Prevotella*-dominated samples, subjects from all three studies predominantly assigned to enterotype 3 (family *Prevotellaceae*-enriched in our designation) showed significantly greater instability (switching between enterotypes, Mann-Whitney test p values 0.024–0.047, Figures 5C and 5D), compared to subjects predominantly assigned to enterotypes 1–2 (enriched in members of the family *Bacteroidaceae*, and also either *Ruminococcaceae* or *Lachnospiraceae*, Figures 5E–5G).

## Discussion

In this study, we utilized a simple model of anaerobic incubation of unprocessed fecal material at 37°C as a proxy for extended colonic retention time. We observed a steady departure from the original composition of both bacteriome and virome, the magnitude of which was dependent on the original microbiome composition. Fecal samples dominated by *Prevotella* (enterotype 3 in this study and enterotype II according to Arumugam et al.^7^) were especially prone to change under these conditions, showing a dramatic reduction of *Prevotella* read counts over time.

Our *in vitro* results complement previous human studies that had demonstrated colonic retention/transit time and linked stool consistency as strong predictors of microbiota composition, with looser stools associated with the *Prevotella* enterotype and firmer stools associated with the *Bacteroides*/*Ruminococcaceae* enterotypes.^11^^,^^15^ Importantly, the *absolute* abundance of *Prevotella* was reported as relatively well conserved across populations, making high *relative* abundance of *Prevotella* a feature typically found in samples with low total microbial loads.^12^ In addition to differences seen in large population cohorts, previous experiment with exposure of a single healthy human fecal microbiota sample to an *in vitro* simulated longer colonic transit time was sufficient to recapitulate some of the microbiota changes seen in elderly and slow transit time patients.^15^ The precise mechanisms responsible for these changes were not fully understood at the time, but our results suggest that the action of bacteriophages, either via prophage induction or attacks of virulent (lytic) phages, may have a significant contribution to depletion of some and overgrowth of other bacterial populations.

Many of the changes in bacterial communities seen in this study as a result of anaerobic fecal incubation, although highly divergent and subject-specific, occurred in parallel with changes in the phage communities. An example of that was the increase in *Crassvirales* and decrease in *Petitvirales* (family *Microviridae*) phages linked to *Prevotella*. The *Crassvirales* comprise a diverse and largely uncultured group of bacteriophages that were previously linked, through a variety of approaches, to bacterial hosts in the phylum Bacteroidota, mainly genus *Bacteroides* (evidence ranging from isolation in culture, single-cell bacterial genomics, and bioinformatics approaches^21^^,^^34^^,^^35^). No *Crassvirales* phages targeting *Prevotella* have been isolated in culture so far; however, an enrichment of a related phage in the presence of *Prevotella in vitro* has been reported.^36^ An increase in overall virome diversity and a tendency toward a decrease in bacterial diversity (insignificant at this sample size) were general features of all samples.

Our findings of rapid parallel changes in bacterial and phage communities in the incubated feces may indicate that bacteria exposed to prolonged residence in a purportedly nutrient-depleted environment might undergo a shift in energy state sufficient to launch prophage induction or to significantly increase sensitivity to virulent phages. These events can potentially cause a dramatic change in microbiota composition, including a shift from enterotype II (*Prevotella*) toward enterotype III (Bacillota) in some samples. Using mice colonized with simple artificial communities, it has been demonstrated that waves of spontaneous prophage induction can lead to dramatic bacterial mortality and change in community structure.^37^ Such events can indeed be triggered by global stress and bacterial SOS response, resulting from increase in transit time, nutrient starvation, and microaeration.^38^^,^^39^

### Limitations of this study

Include: (i) The time gap between voiding and processing of fecal samples and their aerobic exposure. This could have resulted in microbiota composition-dependent oxidative stress and increased mortality in certain taxonomic groups. At the same time, previous studies have shown that raw fecal samples retain good bacterial viability when stored at ambient conditions for up to 24 h.^25^ (ii) The use of 16S rRNA amplicon profiling, as opposed to shotgun metagenomics, to study the composition of bacterial microbiota. This limited our taxonomic resolution to genus level and prevented us from establishing phage-host connections in a more direct fashion (CRISPR spacer matches, prophages in metagenomically assembled genomes etc). (iii) Relatively low number of samples used, which, due to heterogeneity of microbiome compositions could have hindered some common trends in phage-host dynamics that would be more visible in larger donor populations. (iv) The simplified nature of the model and its inability to take into account such covariates of increased colonic residence as nutrient fluxes (including as a result of phage-induced lysis, as well as between the human host and bacteria), changes in gas composition, pH, water absorption etc.

Nevertheless, our data suggests that integrating the analysis of both bacterial and phage communities would provide a more complete understanding of the composition of the bacteriome in feces or in the distal gut, particularly following long colonic residence times. The absence of certain taxa, such as *Prevotella*, could be an accurate reflection of the community structure or could be a result of phage-induced lysis. If the latter was the case, analyzing the bacteriome in isolation would provide a misleading picture of the true bacterial composition. However, the presence of high levels of *Prevotella*-specific phage would confirm that *Prevotella* had been present in significant amounts since every phage is a biomarker of the recent presence of the host bacterium. In order to extend the usefulness of this observation we need to establish more phage-host relationships within the gut microbiome, an effort that would certainly provide better insights into bacterial structure in the microbiome.

## STAR★Methods

### Key resources table

**Table:** 

REAGENT or RESOURCE	SOURCE	IDENTIFIER
**Chemicals, peptides, and recombinant proteins**		

Anaerocult™ A anaerobic pouches	Merck Millipore	Cat#1323810001
Glass Homogenisation Beads 3.5mm	Biospec Products	Cat#11079135
Zirconium Silicate Beads 0.1mm	Thistle Scientific	Cat#11079101z
Zirconium Silicate Beads 1mm	Thistle Scientific	Cat#11079110z
AMPure XP beads	Beckman-Coulter	Cat#A63881
Oxoid Maximum Recovery Diluent	ThermoFisher Scientific	Cat#CM0733
Oxoid Bile Aesculin Agar	ThermoFisher Scientific	Cat#CM0888
Oxoid MRS Agar	ThermoFisher Scientific	Cat#CM1153
Oxoid Columbia Blood Agar	ThermoFisher Scientific	Cat#CM0331
Oxoid RCM Agar	ThermoFisher Scientific	Cat#CM0151
LightCycler® 480 SYBR Green I Master	Roche	Cat#04707516001

**Critical commercial assays**		

DNeasy Blood & Tissue kit	QIAGEN	Cat#69506
QIAamp Fast DNA Stool Mini kit	QIAGEN	Cat#51604
Qubit dsDNA HS kit	ThermoFisher Scientific	Cat#Q32854
illustra GenomiPhi V2 DNA Amplification Kit	GE Healthcare Life Sciences	Cat#25660032
Nextera XT DNA Library Preparation Kit	Illumina	Cat#FC-131-1096
Agilent High Sensitivity DNA Kit	Agilent	Cat#5067-4626
Nextera XT Index kit v2 Set A	Illumina	Cat#FC-131-2001
Nextera XT Index kit v2 Set B	Illumina	Cat#FC-131-2002
Nextera XT Index kit v2 Set C	Illumina	Cat#FC-131-2003
Nextera XT Index kit v2 Set D	Illumina	Cat#FC-131-2004

**Deposited data**		

Raw sequencing data	NCBI BioProject	PRJNA905467
Viral RefSeq database release 208	NCBI FTP	ftp.ncbi.nlm.nih.gov/refseq/release/viral/
MGV database	Nayfach et al., 2021^40^	https://doi.org/10.1038/s41564-021-00928-6
GPD database	Camarillo-Guerrero et al., 2021^29^	https://doi.org/10.1016/j.cell.2021.01.029
IMG/VR3 database (release 2020-10-12_5.1)	Roux et al., 2021^41^	https://doi.org/10.1093/nar/gkaa946
pVOGs database	Grazziotin et al., 2017^42^	https://doi.org/10.1093/nar/gkw975
PHROGs database	Terzian et al., 2021^43^	https://doi.org/10.1093/nargab/lqab067
ICTV virus metadata resource (VMR)	International Committee on Taxonomy of Viruses	https://ictv.global/vmr

**Oligonucleotides**		

PCR primer sequences are given in the STAR Methods section.	Clifford et al., 2012^44^; Matsuki et., al 2002^45^; this study.	NA

**Software and algorithms**		

USEARCH v8.1	Edgar, 2010^46^	https://doi.org/10.1093/bioinformatics/btq461
RDP Classifier v2.12	Wang et al., 2007^47^	https://doi.org/10.1128/AEM.00062-07
Trimmomatic v0.36	Bolger et at., 2014^48^	https://doi.org/10.1093/bioinformatics/btu170
SPAdes v3.13	Nurk et al., 2017^49^	https://doi.org/10.1101/gr.213959.116
ViromeQC v1.0	Zolfo et al., 2019^50^	https://doi.org/10.1038/s41587-019-0334-5
VirSorter2 v2.2.4	Guo et al., 2021^51^	https://doi.org/10.1186/s40168-020-00990-y
Prodigal v2.6.3	GitHub	https://github.com/hyattpd/Prodigal
HMMER v3.1b1	http://hmmer.org/	NA
CheckV v1.0.1	Nayfach et al., 2021^40^	https://doi.org/10.1038/s41587-020-00774-7
vConTACT2	Jang et al., 2019^52^	https://doi.org/10.1038/s41587-019-0100-8; https://bitbucket.org/MAVERICLab/vcontact2/src/master/
BACPHLIP	Hockenberry & Wilke, 2021^53^	https://doi.org/10.7717/peerj.11396
R environment v4.3.2	https://www.r-project.org/	NA
ggplot2 v3.4.3	https://cran.r-project.org/	NA
phyloseq v1.44	https://www.bioconductor.org/	NA
vegan v2.6-4	https://cran.r-project.org/	NA

### Resource availability

#### Lead contact

Further information and requests for resources and reagents should be directed to and will be fulfilled by the lead contact, Andrey Shkoporov (andrey.shkoporov@ucc.ie).

#### Materials availability

This study did not generate new unique reagents.

#### Data and code availability

Raw sequencing data (bacterial 16S rRNA amplicon sequencing and viral DNA shotgun sequencing) is available from NCBI SRA database under NCBI BioProject: PRJNA905467 (BioSample accession numbers are listed in Table S1). OTU tables, 16S OTU sequences, assembled viral contigs and taxonomic assignments are available in the accompanying supplementary dataset. This study does not report original new code. Any additional information required to reanalyze the data reported in this paper is available from the lead contact upon request.

### Experimental model and study participant details

#### Collection and incubation of fecal samples

Fecal samples were collected between 2016 and 2017 from 13 healthy men and women (UCC employees and students, residents of Cork, Ireland), in accordance with study protocol APC055^33^ approved by the Cork Research Ethics Committee. Samples were anonymized by APC Microbiome Ireland research nurses and issued with unique subject-specific identifiers (Table S1). One of the subjects (916) was sampled twice. More extensive participant metadata is available from the original cohort study publication.^33^ Samples were collected in participants homes and delivered to the lab in tightly closed containers within 2–3 h after voiding. Immediately upon delivery samples were aseptically aliquoted into 1 g portions (in triplicates) and placed into individual Sterilin containers. Samples were then incubated anaerobically with Anaerocult A pouches in anaerobic jars (Merck Millipore) at 37°C and frozen at −80C at the indicated time points.

### Method details

#### Extraction and shotgun sequencing of VLP-associated DNA

Fecal VLP-associated DNA was extracted from 0.5 g of fecal material after thawing, as described before.^28^ Fecal DNA was subjected to whole-genome multiple displacement (MDA) amplification and sequencing libraries were prepared using a tagmentation approach (Nextera XT Library Preparation kit, Illumina) in accordance with the previously published protocols.^28^^,^^36^ Libraries were assessed using Bioanalyzer High Sensitivity DNA assay (Agilent) and sequenced on an Illumina HiSeq 2500 platform at GATC Biotech AG, Germany. Raw sequencing data is available from NCBI SRA database under NCBI BioProject: PRJNA905467 (Table S1).

#### Bacterial microbiota profiling

Total microbial DNA was extracted from 200 mg of thawed fecal sample. DNA extraction, amplification of V3-V4 hypervariable region of 16S rRNA gene were performed as described before.^17^^,^^28^ Reads were processed using USEARCH v8.1 pipeline.^46^ Reads were merged and filtered (usearch -fastq_mergepairs and usearch -fastq_filter) by expected error rate of 0.5 and targeting merged sequence size of 400–475 bp. Forward (17 bp) and reverse (21 bp) primer sequences were removed (usearch -fastx_truncate). Resulting sequences were dereplicated (usearch -derep_fulllength) and singletons were removed (usearch -derep_fulllength). OTUs were picked by clustering dereplicated sequences to 97% identity (usearch -cluster_otus), followed by reference -based chimera removal (usearch -uchime_ref). OTU table was constructed by mapping merged and filtered reads to picked OTUs (usearch -usearch_global followed by an accompanying script “uc2otutab.py”). Taxonomy was assigned using a pretrained RDP Classifier v2.12.^47^

#### Bioinformatic analysis of VLP metagenomic data

Illumina reads were trimmed, filtered using Trimmomatic v0.36 and assembled using MetaSPAdes v3.13.0 into contigs on a per-sample basis as described before.^17^^,^^36^ Contigs >1 kb in length were dereplicated with a BLASTn cut-off of 90% nucleotide identity over 90% of length to keep the longest representative contig of each cluster.^36^ Levels of bacterial DNA contamination in raw reads before assembly were assessed using ViromeQC.^50^

Viral genomic contigs were selected from background bacterial and host DNA contamination using the following set of steps. VirSorter2^51^ was used as a primary tool to identify possible viral contigs (databases used: dsDNAphage, RNA, ssDNA). Additionally, BLASTn search (*e*-value cut-off 1e-10) against viral RefSeq (release 208), MGV,^30^ GPD,^29^ GVD,^54^ IMG/VR3^41^ (release 2020-10-12_5.1) databases, as well as our in-house database of *Crassvirales* genomes to identify homologs of known viruses. Protein-coding genes on contigs were predicted using Prodigal v2.6.3 and annotated using hmmscan search (HMMER 3.1b2, *e*-value cut-off 1e-05) against viral protein family HMM databases pVOGs^42^ and PHROGs.^43^ Completeness of viral genomic contigs was assessed using CheckV.^40^ To be deemed as viral assembled contigs should satisfy the following criteria: (1) have ≥50% nucleotide identity over ≥85% of the contig length to a known virus; or (2) have strong VirSorter2 viral assignment – score ≥0.9 OR score ≥0.7 with at least 1 viral hallmark protein-coding gene present; and (3) not be classified as a provirus by CheckV. Using this approach 12,828 complete and partial viral genomic contigs were identified across all samples.

Contigs were grouped into viral clusters (VCs) using vConTACT2 pipeline^52^ utilizing BLASTp-based MCL clustering of viral proteins and ClusterONE mode for VCs. Bacterial hosts for phage contigs were predicted by combining host assignments from hits to an in-house database^17^ of bacterial CRISPR spacers(*e*-value cut-off 1e-05, bitscore cut-off 65) and existing host annotations of closely related phage genomic contigs (species-level sequence similarity, ≥95% BLASTn nucleotide identity over ≥85% of contig length) in the IMG/VR3 database.

Taxonomic classification of contigs was performed first by finding close relatives (species-level, ≥95% BLASTn nucleotide identity over ≥85%of contig length) to contigs among the exemplar genomes of viral taxa recognized by the International Committee on Taxonomy of Viruses (ICTV, list of GenBank accession numbers available at https://ictv.global/vmr), where available and/or among entries of the IMG/VR3 database. Next, genus-level annotations from ICTV (where available), and family level annotations from IMG/VR3 were extrapolated to all other members of the respective VCs, lacking direct hits to ICTV and IMG/VR3 genomes. Phage lifestyle (temperate or virulent) was predicted using BACPHLIP.^53^ Viral contig properties are summarized in Table S2.

Finally, in order to assess relative abundance of individual contigs in the viromes, trimmed and filtered Illumina reads were aligned to selected viral contigs (see above) and the count matrix was produced as described before.^8^ Cases of sparsely covered contigs (less than 75% of contig coverage with at least 1x density) were filtered out. Viral genomic contigs, read counts and breadth of coverage data are available in the accompanying Dataset.

#### Bacterial cultures

Fecal samples were serially 10-fold diluted in Oxoid Maximum Recovery Diluent (ThermoFisher) and plated onto Oxoid Bile Aesculin Agar (BEA, enumeration of enterococci), De Man-Rogosa-Sharpe agar (MRS, enumeration of lacobacilli), Columbia agar with 5% defibrinated sheep blood, 0.01 g/L hemin, 0.01 g/L vitamin K (enumeration of a range of anaerobic bacteria), and Reinforced Clostridial (RCM) agar supplemented with 5 g/L propionic acid, 2.5 g/L lactulose and 0.01 g/L riboflavin (enumeration of bifidobacteria). Plates were incubated for 48–72 h anaerobically with Anaerocult A pouches in anaerobic jars (Merck Millipore) at 37°C, and colonies were counted. Representative colony types were subjected to Gram staining and streaked out in aerobic and anaerobic conditions to confirm approximate identity of the isolates. As expected, gram-positive facultative anaerobic cocci were recovered from bile aesculin agar, gram-positive facultative anaerobic rods were seen on MRS agar. Columbia agar produced a variety of mostly gram-negative strictly anaerobic rods consistent with *Bacteroidales* order morphology. Isolates from supplemented RCM agar were mostly irregular and bifid-shaped gram-positive rods.

#### qPCR

Quantitation of total bacterial DNA was performed using Light Cycler 480 (Roche), LightCycler PCR Master SYBR Green I kit and other standard Light Cycler consumables (384-well plates with sealing foils). The following PCR program was used in all assays: initial denaturation at 95°C 3 min, followed by 40 cycles of 95°C 20 s, 60°C 20 s, 72°C 30 s.

Universal bacterial 16S rRNA gene primers (U16SRT-F 5′-ACTCCTACGGGAGGCAGCAGT-3’; U16SRT-R 5′-TATTACCGCGGCTGCTGGC-3′) were described previously.^44^ Bacteria of the genera *Bacteroides* and *Prevotella* were quantified using primers (g-Bfra-F 5′-ATAGCCTTTCGAAAGRAAGAT-3’; g-Bfra-R 5′-CCAGTATCAACTGCAATTTTA-3’; g-Prevo-F 5′-CACRGTAAACGATGGATGCC-3’; g-Prevo-R 5′-GGTCGGGTTGCAGACC-3′) described by Matsuki et al.,^45^ genus *Bifidobacterium* and species *Escherichi coli* were detected using primers Bif-xfp-F1 5′-CGTCCGTTCTACCCGATG-3′ Bif-xfp-R1 5′-GGTCTTCTTGCCGTCGAT-3′ Eco-uidA-F1 5′-CTCTTTAGGCATTGGTTTCG-3′ Eco-uidA-R1 5′-TTGCTGAGTTTCCCCGTT-3′, targeting *Biffidobacterium* spp. xylulose-5-phosphate/fructose-6-phosphate phosphoketolase and *E*. *coli* beta-glucuronidase genes respectively. Total community DNA extracted as described before^17^ was used as template. DNA extracted using the same method from standardized quantities (10^9^ cells) of bacterial strains was used as calibration standard. Individual phage genomes were assayed in both total community DNA and VLP DNA prepared as described above using the following primer pairs: FD1-node11-F1 5′-ACCTTGTTATAATGGTCGTGGT-3’; FD1-node11-R1 5′-AGCAGCTATTGCAGCATGTC-3’; FD1-node2-F1 5′-CGTCTTGTTGTTTCCCTTGCC-3’; FD1-node2-R1 5′-CCGCATTCACTACATCTCCA-3’; FD1-node2164-F1 5′-GTGCTGTTTCTCCGATTGTC-3’; FD1-node2164-R1 5′-ACCTGTAGTCTCTTCGACCA-3’.

### Quantification and statistical analysis

Statistical analysis of sequencing data was carried out in R environment v4.3.2. Visualizations were created using ggplot2 v3.4.3. Bacterial 16S and viral shotgun metagenomics data were handled as phyloseq (v1.44) objects. Bacterial OTUs with less than 3 reads aligned in any of the samples, as well as viral OTUs with less than 10 reads aligned were discarded from the dataset. Alpha-and beta-diversity metrics were computed in phyloseq: phyloseqplot_richness(); phyloseqordinate(x, "PCoA", "bray"); phyloseqplot_ordination(). Spearman correlations between relative abundances of bacterial/viral OTUs (contigs) and time of incubation (Figure 3, Table S3) were calculated on a per-subject basis (for each subject/fecal sample separately), followed by Benjamini-Hochberg correction of p values. Enterotype clustering was performed using k-means (k = 3) algorithm. Enterotype switching (“diversity” of enterotype states in a given subject) was calculated as Simpson diversity index [vegandiversity() in vegan v2.6-4].

## Supplemental information

Document S1. Figures S1–S5

Table S1. Biological sample metadata, related to Figures 1, 2, and 4

Table S2. Assembled viral contig metadata, related to Figures 2 and 4

Table S3. Viral contig / bacterial 16S OTU correlation data, related to Figure 3

Dataset S1. Viral genomic contig sequences, viral contig read counts and coverage matrices, 16S OTU sequences, 16S OTU read count matrix, 16S OTU taxonomy table, related to Figures 1–4

## Footnotes

Supplemental information can be found online at https://doi.org/10.1016/j.isci.2023.108778.
