# Supplementary material for: Dynamic nature of viral and bacterial communities in human faeces
Source: iScience. 2023 Dec 29;27(2):108778. doi: 10.1016/j.isci.2023.108778 (PMC10825054; doi:10.1016/j.isci.2023.108778)
Supplement: Document S1. Figures S1–S5 [file mmc1.pdf]

iScience, Volume *27*

Supplemental information

Dynamic nature of viral and bacterial communities in human faeces

Andrey N. Shkoporov, Orla O'Regan, Linda Smith, Ekaterina V. Khokhlova, Lorraine A. Draper, R. Paul Ross, and Colin Hill

Supplemental Figures

Fig. S1. Virome sequencing and assembly. Related to Fig. 1. **A**, size, coverage and taxonomic distribution of 12,828 viral genomic contigs recovered from all 14 faecal samples in this study; **B**, ViromeQC scores reflecting degree of virome enrichment (compared to presumed bulk metagenome) across all time points of incubation in all samples; boxplots are standard Tukey type with interquartile range (box), median (bar) and Q1 – 1.5 × IQR/Q3 + 1.5 × IQR (whiskers). **C**, concentration of extracted VLP DNA across time points (horizontal bars represent median values); **D**, concentration of extracted total community DNA across time points (horizontal bars represent median values).

Fig. S2. Virome (A) and bacteriome (B) α-diversity evolution over the course of anaerobic incubation of faecal samples. Related to Fig. 2. Values given are Pearson correlation of diversity metrics with time of incubation. Linear regression lines are show along with 95% CI.

Fig. S3. Gradual departure of bacteriome and virome composition from the original state, visualised Bray-Curtis dissimilarity metric (β-diversity). Related to Fig. 2. **A**, Kruskal-Wallis test followed by post hoc Mann-Whitney pairwise tests; **B**, Person correlation and linear regression with 95% CI.

Fig. S4. PCoA ordination of Bray-Curtis distances between bacteriome (A) and virome (B) samples from eight individuals. Related to Fig. 2. Numbers next to data points are incubation time in hours. Confidence ellipsoids represent 95% CI.

Fig. S5. Results of culture-based and qPCR quantification of some bacterial groups in six incubated faecal samples. Related to Fig. 4. BEA, bile aesculin agar (enterococci); Columbia, Columbia blood agar (gram-negative anaerobes); MRS, de Man-Rogosa-Sharpe agar (bifidobacteria and lactobacilli); RCM, reinforced clostridial agar (gram-positive anaerobes). Filled circles are median values, vertical bars show range between minimum and maximum value across three technical replicates. See Methods section for details.

Figure S1

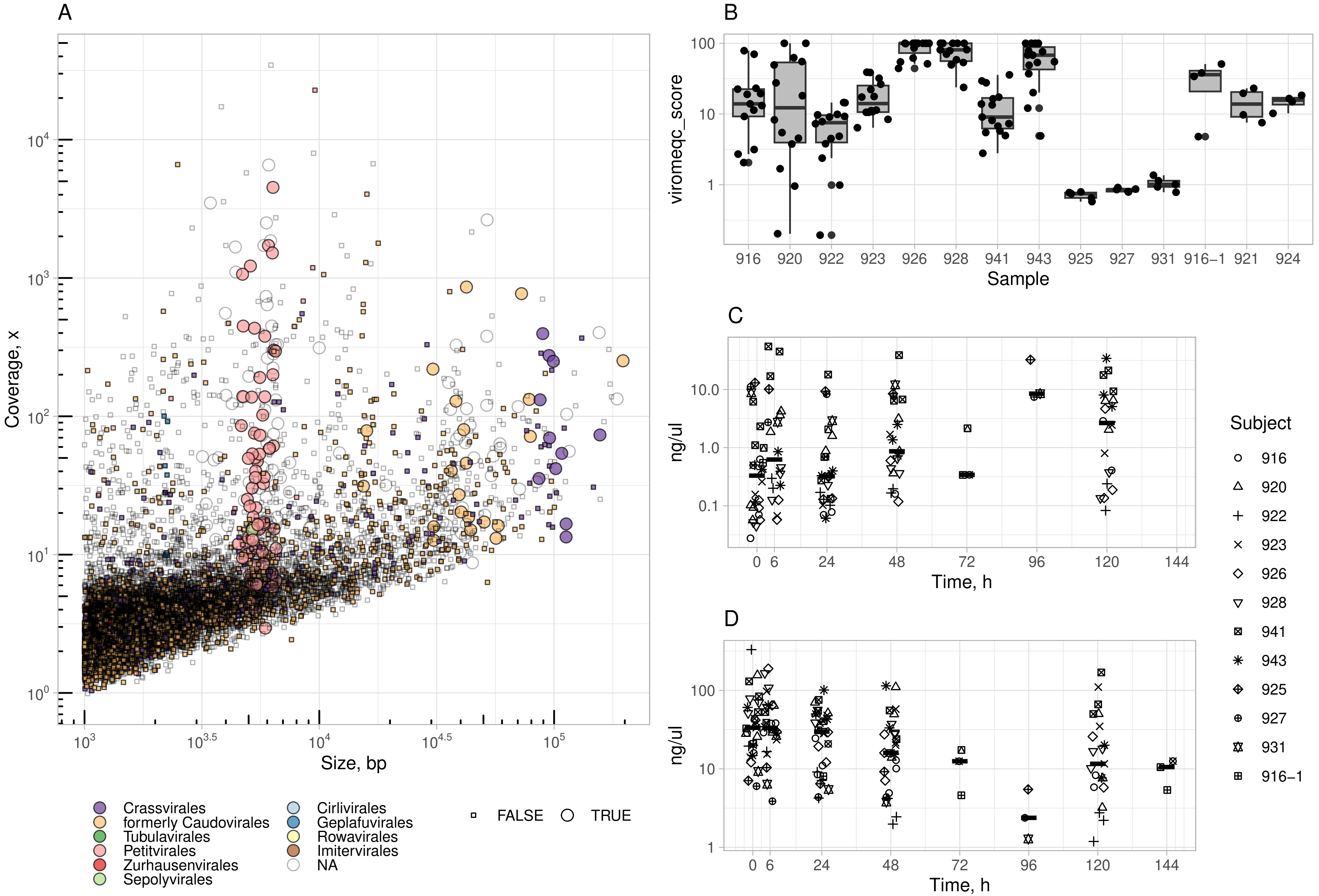

A
Coverage, x
Size, bp
10^0
10^1
10^2
10^3
10^4
10^3
$10^{3.5}$
10^4
$10^{4.5}$
10^5
Crassvirales
formerly Caudovirales
Tubulavirales
Petitvirales
Zurhausenvirales
Sepolyvirales
Cirlivirales
Geplafuvirales
Rowavirales
Imitervirales
NA
FALSE
TRUE
B
viromeqc_score
Sample
100
10
1
916
920
922
923
926
928
941
943
925
927
931
916−1
921
924
C
ng/ul
Time, h
10.0
1.0
0.1
0
6
24
48
72
96
120
144
D
ng/ul
Time, h
100
10
1
Subject
916
920
922
923
926
928
941
943
925
927
931
916−1

Figure S2

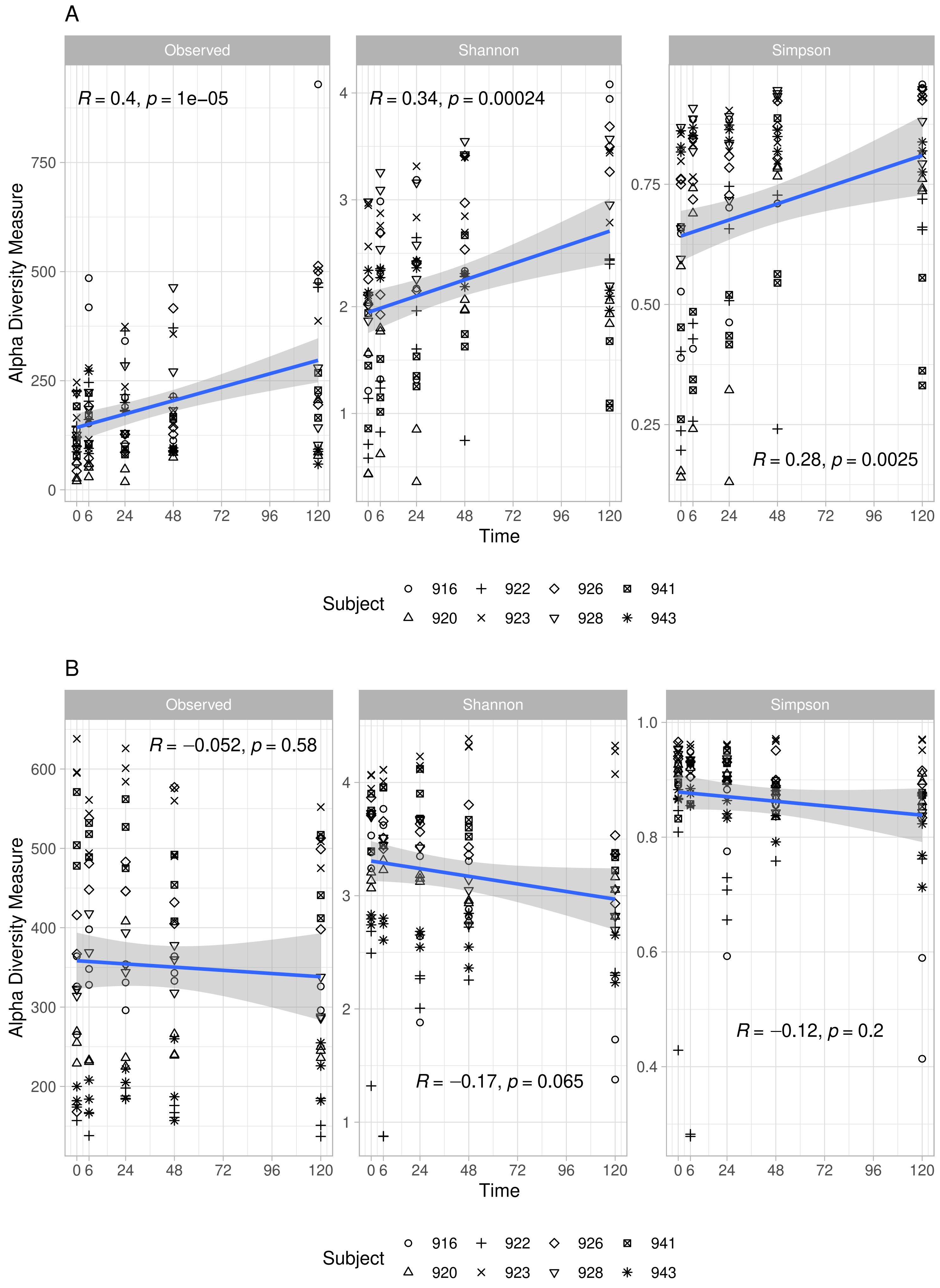
A
Observed
Shannon
Simpson
R = 0.4, p = 1e−05
R = 0.34, p = 0.00024
R = 0.28, p = 0.0025
Alpha Diversity Measure
Time
Subject
916
920
922
923
926
928
941
943
B
Observed
Shannon
Simpson
R = −0.052, p = 0.58
R = −0.17, p = 0.065
R = −0.12, p = 0.2
Alpha Diversity Measure
Time
Subject
916
920
922
923
926
928
941
943

Figure S3

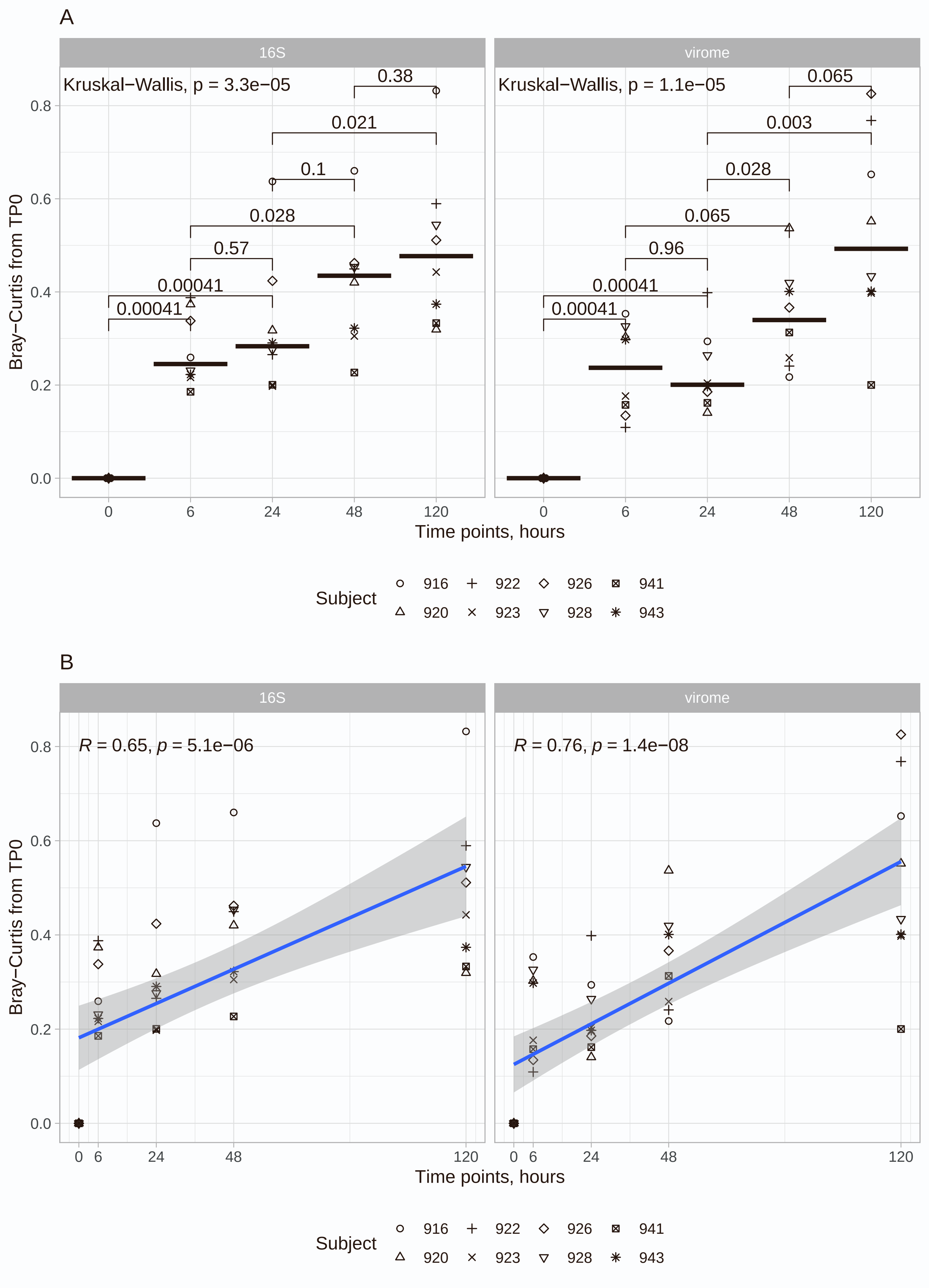
A
16S
virome
Kruskal−Wallis, p = 3.3e−05
Kruskal−Wallis, p = 1.1e−05
0.38
0.021
0.1
0.028
0.57
0.00041
0.00041
0.065
0.003
0.028
0.065
0.96
0.00041
0.00041
Bray−Curtis from TP0
Time points, hours
Subject
916
922
926
941
920
923
928
943
B
16S
virome
R = 0.65, p = 5.1e−06
R = 0.76, p = 1.4e−08
Bray−Curtis from TP0
Time points, hours
Subject
916
922
926
941
920
923
928
943

Figure S4

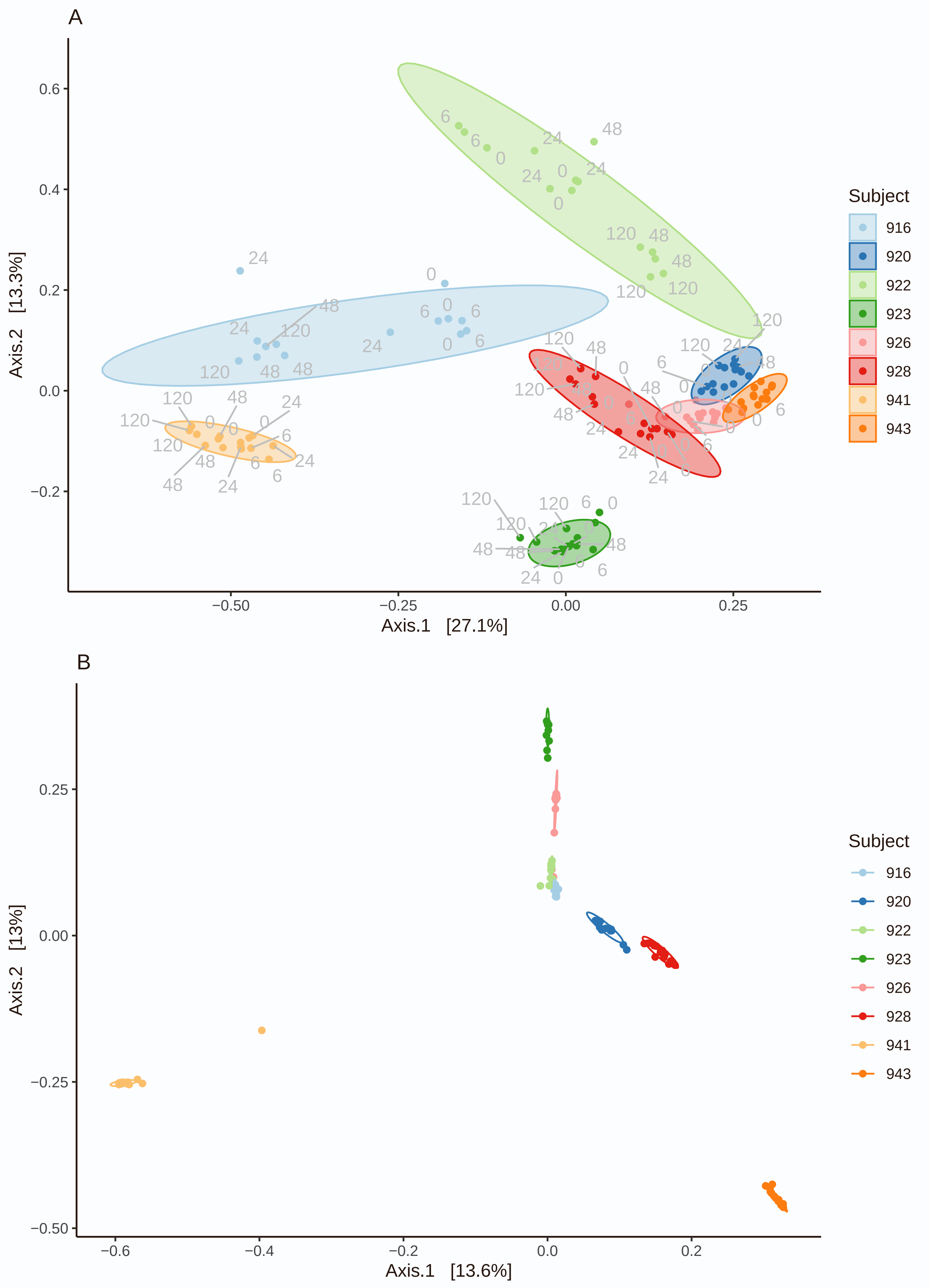
A
Axis.1 [27.1%]
Axis.2 [13.3%]
−0.50
−0.25
0.00
0.25
0.6
0.4
0.2
0.0
−0.2
Subject
916
920
922
923
926
928
941
943
B
Axis.1 [13.6%]
Axis.2 [13%]
−0.6
−0.4
−0.2
0.0
0.2
0.25
0.00
−0.25
−0.50
Subject
916
920
922
923
926
928
941
943

Figure S5

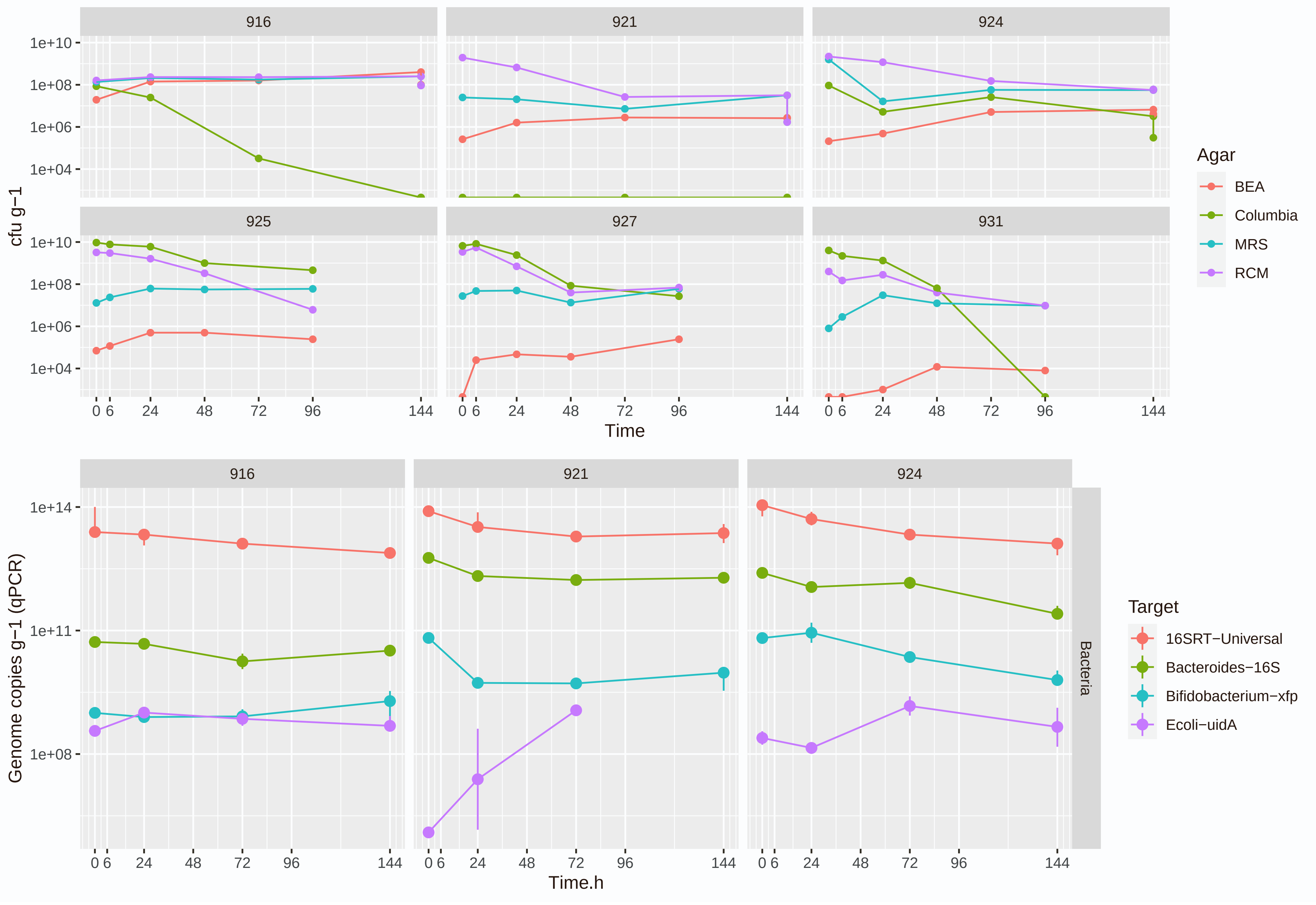

916
921
924
925
927
931
1e+10
1e+08
1e+06
1e+04
cfu g−1
0 6
24
48
72
96
144
Time
Agar
BEA
Columbia
MRS
RCM
1e+14
1e+11
1e+08
Genome copies g−1 (qPCR)
Time.h
Bacteria
Target
16SRT−Universal
Bacteroides−16S
Bifidobacterium−xfp
Ecoli−uidA
